# Pharmaceutical Processes

**Published:** 1800-01

**Authors:** 


### *Pharmaceutical Proceſſes.*

Cit. Bouillon Lagrange read a Memoir on the Analyſis of the *Caſſia ſenna*, of Linnæus.—A great number of experiments on the leaves of this plant, which is frequently uſed in medicine, have convinced the author, that his analyſis differed little from that of the bark. He thinks, that the ſame reſult will take place with all dry vegetable ſubſtances treated in a ſimilar manner, and that ligneous vegetables produce the ſame effects when treated with the ſame re-agents; ſo that one analyſis of this kind well performed may ſerve as a precedent for all others of an analogous nature. As to the manner of employing the ſenna, Cit. Lagrange, in every inſtance, adviſes a cold infuſion to be made, and not to combine it with acid tinctures and ſpirituous liquids, which change the nature of this medicine by oxygenating the ſaponaceous principle, which, by that means, acquires acquires the nature of refins. This change makes it act differently on the animal œconomy; it then occafions violent colics without purging, while in the former cafe it purges without griping.—*Rapport Général des Travaux de la Société Philomatique de Paris.* Vol. I. p. 53.

---

Prof. Lichtenstein, propofes the following method of preparing nitric æther, *without the application of fire.* Having placed a tubulated retort in fnow, or cold water, with a proportionate receiver faftened by a foft bladder, and provided the apparatus with an air tube, you fill it with three parts of alcohol, and gradually add two parts of nitrous acid; you then clofe the retort with a glafs ftopper, tied on with a wet bladder. The mixture foon begins to effervefce and boil, air bubles are difengaged, and fill the receiver with a vapour, which, when condenfed into a fluid, yields nitric æther. The precaution of furnifhing the apparatus with an air tube, is, on account of the elaftic fluid difengaged during the procefs, always neceffary, to fecure the operator from the danger of the veffel being blown to pieces; a circumftance which is likewife confirmed by the experiments of Prof. Göttling, *Berlin. Iahrbuch für die Pharmacie*, B. III. p. 222.

---

Prof. Lowitz has difcovered a very eafy method of *depriving æther of its alcohol*, which will prevent the neceffity of its frequent rectification, always attended with lofs. On pouring the æther charged with alcohol upon dry muriat of lime, and fufficiently agitating the whole, the alcohol is abforbed, and the æther brought to fuch purity and perfection, that its fpecific gravity at the temperature of + 20° Reaum. is =732. If it be a fecond time diftilled over dry muriat of lime, it will fink even to 716. During this rectification, the æther arifes in the form of gas, and the receiver ought to be cooled with fnow water, or ice broken fmall, in order to condenfe it into drops. *Gren's Journal*, B. III. p. 313.

---

The method of *rendering alkalis cauftic*, adopted by Prof. Trommsdorf, is, on account of its facility, worthy of our attention. To the boiling alkaline folution, he gradually adds finely powdered quick lime, till the whole is perfectly faturated. The liquor is then filtered, and fubmitted to the ufual mode of treatment. The advantage of this procefs is, that the lime does not fo readily effervefce, as when flacked in water, fo that the feparation of the alkaline ley is much more eafily effected.—*Pharmaceut. Experimental Chemie*, p. 146.

---

Mr.

Mr. Kirchhoff has, according to the directions of Prof. Lowitz, completely decomposed the oxyd of tungsten, *via humida*, by means of carbonat of pot-ass. He incessantly triturated a fine powder of the mineral, with a concentrated solution of the salt, till the mixture assumed the consistence of a liniment; and he afterwards exposed it to a moderate heat for digestion. Prof. Lowitz promotes the process, by nearly evaporating a mixture of 1½ lb. of oxyd of tungsten, and 2 lb. of vegetable alkali with 3 lb. of water, at four successive times, while the whole must be continually stirred. The muriatic acid yielded, during the solution of the barytes, only 6 drachms of residue; a convincing proof of the decomposition of the oxyd. *Journal der Pharmacie*, B. iii. p. 354.

---

Although Prof. Trommsdorf does not entirely approve of the mode adopted by Van Mons for the preparation of magnesia, yet we are induced to recommend it as advantageous to the attentive observer; we shall therefore give the following account of the process. A solution of the sulphat of magnesia and muriat of soda is placed in a cellar, and left undisturbed, till a complete decomposition of the particles has taken place; from which, sulphat of soda and muriat of magnesia are produced. Carbonat of lime is then added, to effect a new decomposition, of which muriat of lime and carbonat of magnesia are the result; the latter in the form of crystals. That the magnesia may not be intermixed with silicious earth, it is necessary to use calcined oyster shells. As, however, the just proportion of these substances ought to be previously ascertained, which, as Prof. Trommsdorf justly remarks, is attended with some difficulty, this process can be recommended only to the experienced operator.—*Journal der Pharmacie*, B. III. p. 277.

*Medical*

